# A Disposable Diaper Collection Project in Langa, Cape Town, South Africa: A Pilot Study

**DOI:** 10.3390/ijerph21101292

**Published:** 2024-09-27

**Authors:** Catherina Schenck, Hugh Tyrrell, Lizette Grobler, Rissa Niyobuhungiro, Alexander Kimani

**Affiliations:** 1 DSI/NRF/CSIR Chair in Waste and Society, University of the Western Cape, Bellville 7535, Western Cape, South Africa; lizziegrob@gmail.com (L.G.); 3987095@myuwc.ac.za (R.N.); 2 GreenEdge Consulting, Cape Town 7708, Western Cape, South Africa; info@greenedge.co.za; 3 Department of Geography, Environmental Studies and Tourism, University of the Western Cape, Bellville 7535, Western Cape, South Africa; 3535526@myuwc.ac.za

**Keywords:** disposable diapers, nappies, collection models, early childhood centres, sustainable development goals

## Abstract

In developing countries, there is currently no established waste management plan that includes resource recovery from used disposable diapers (DDs) apart from incineration and landfilling. In low-income areas with limited storage space, the complex composition and odour of used DDs make it impossible to manage properly if not supported by effective waste management systems. In the absence of effective waste management, DDs are dumped in open spaces, burned or buried. These actions pose threats to the safety and health of humans, animals and the environment. Separation and collection of DDs are critical preliminary steps to landfilling, recycling or beneficiation. In this article, we describe a case study of two pilot collection projects in Langa township in Cape Town, South Africa, to determine whether and how a source-separated collection system can work in low-income, resource-constrained areas. The lessons learned highlighted the following: The eagerness of parents to participate for the benefit of their own and their children’s health; the complementarity of the two pilot collection models to serve the needs of the community; the important role non-government organisations play in the implementation of waste management projects; the significance of the possible job creation opportunities and the unintended benefits of enhancing social cohesion. The financial sustainability of these projects needs further exploration.

## 1. Introduction

The emergence of absorbent hygiene products (AHP) is generally held to have contributed to improving the quality of life of millions of people through better hygiene, skin protection, and convenience [1]. Disposable diapers (DDs) present the largest percentage of the AHP market [2]. Globally, an estimated 90–95% of parents use disposable diapers (DDs) as they are believed to be comfortable, hygienic, cost-effective, convenient and beneficial to infants [2,3,4,5,6,7]. In low- or middle-income countries (LMICs) in the Southern African Development Community (SADC) region, the percentages are lower. In Kenya, for example, estimates of caregivers using DDs range from 86 to 95%, while 61 to 78% of caregivers opt for DDs in Zimbabwe [8]. Studies in South Africa [6,7,9] mirror global consumption patterns.

Despite its contribution to the improvement of global sanitation conditions, the increase in consumption of AHP products in general, and DDs specifically, poses an escalating environmental challenge [8,10]. Moreover, research on these issues is limited [8,10]. Salient themes emerging from the recent literature are the difficulty in handling used DDs, its mismanagement and detrimental environmental impact [6,7,8,11].

In developing countries, according to Tsigkou [12], there is currently no established waste management plan that includes resource recovery from used disposable nappies (DDs), apart from incineration and landfilling. These two alternatives present serious downsides. In developing and resource-constrained countries and communities, the complex composition of DDs makes it impossible to manage properly if not supported by effective waste management systems [6,9,13]. A typical unused DD comprises (i) nonwoven fabrics (mainly polypropylene (PP) and polyethylene (PE)) that make up the inner, outer, and acquisition and distribution layer (ADL), (ii) sodium polyacrylate, playing the role of super absorbent polymer (SAP), and finally (iii) cellulosic fibres (or fluffy pulp) that alongside SAP make up the absorbent core [1,12,14]. Tsigkou et al. [12] regard the composite nature of the DD as the most important hindrance in implementing an appropriate treatment or resource recovery process [12,13,14,15]. Currently, without treatment plans, a DD is worth nothing, but the entire life cycle of these items results in an annual emission of 2.7 million t of greenhouse gases [15]. The proliferation of landfilled DDs poses a number of global environmental concerns, as their decomposition time is estimated to be 200 to 500 years [2,4,8,16,17]. The impact is even worse in countries and communities where waste management is limited, inefficient or absent, and DDs are dumped, burned and buried [6,9,13,17,18,19].

### Background to the Study and Problem Statement

In 2023, the South African government’s Department of Fisheries, Forestry and the Environment (DFFE) published a proposed policy and strategy document for the design and disposal of AHP waste in South Africa. It is estimated that between 700–900 tonnes of AHP products are used in the country. Most AHP products are landfilled by municipalities or, in the absence of effective waste management, are dumped in open spaces or the natural environment and burned or buried. These actions pose threats to the safety and health of humans, animals and the environment [6,7,9,18,19,20]. Consequently, the appropriate design and management of AHP is imperative.

However, management of AHPs, particularly the disposal of DDs, presents various challenges in terms of collection and transportation. Due to the low value-to-weight ratio of post-consumer AHPs, the collection and transportation of waste AHPs over long distances is not preferred or economical. Therefore, AHP disposal options are best located close to the source of the bulk waste [21].

Several disposal options were assessed to determine, amongst other things, their suitability for AHP processing, technology readiness, flexibility to handle other residual waste, processing capacity and price. Six of these technology options appeared to meet the project scope, i.e., sustainable disposal or repurposing of AHPs. These solutions include technologies offered or employed by FaterSmart sPa (Roma, Italy), Knowaste (Toronto, Canada), Super Faiths Diaper (SFD) (Tokyo, Japan), Pyrocore (Bristol, UK), Asher (Kuala Lumpur, SA/Malaysia) and Unicharm (Tokyo, Japan). These disposal solutions treat most (if not all) of the AHP raw material and contents, provided there is a robust collection system in place [20].

As a consequence of the South African government’s policy shift, its investigation of disposal options and the recognition of the significance of adequate collection systems, this study reports the results and conclusions of a research project with the aim of determining whether and how source-separated collection systems can work in low income, resource-constrained areas.

## 2. Literature Review

Separation and collection of DDs are critical preliminary steps to landfilling, recycling or beneficiation [22] but have received scant attention until recently. Although some studies have focused on the willingness of caregivers to participate in the collection of DD waste in Nairobi, Kenya [23] and West Jakarta [24], very few research studies have focused on collection systems.

The most recent collection system case studies were conducted in Korea and Scotland. Kim and Kim [4] confirm that the Korean case study builds on conclusions regarding the optimisation of the collection and transportation of diaper waste from a 2012 Scotland collection trial. Here, participants were offered a choice between a curbside or pick-up collection service using containers provided for diaper waste or using recycling sacks with a tie that they could transport to the Households Waste Recycling Center (HWRC). The weekly curbside collection service was preferred over the participant-initiated transport and resulted in a higher opt-in rate, public satisfaction and tonnage of collected waste. Moreover, it had a lower cost per ton.

The most salient factors relating to the optimal collection of diapers from the Scotland collection trail were the willingness to participate in collection initiatives (“opt-in rate”) and the contribution of strategic communication measures to increase participation [4]. These included advertising materials and direct community engagement with participants, emphasising the environmental benefits of diaper collection. Another key aspect was the necessity to provide containers of adequate capacity.

The Korean study was based on the findings of the Scotland trial, and a similar collection method with significant variations was applied [4]. These included container capacity, container format, participant pool, collection point, collection frequency and communication and engagement approach. While the most efficient collection method in the Scotland trial incorporated containers and 30 L sacks [4], the Korean study included 16 L indoor collection boxes and 20 L waste bags. Containers were also placed outside the daycare centres.

Regarding the pool of participants, the Scotland trial included households with children or other adults with incontinence needs who used diapers [4]. The Korean trial focused on larger units: daycare centres in the Nowon-Gu district of Seoul. This choice was justified due to greater diaper quantities and transport convenience.

Collection frequency was weekly in the case of the curbside collection in the Scotland trial, while collection points at the daycare centres in the Korean trial were serviced three times per week [4].

Finally, in terms of communication and engagement, advertising materials such as leaflets were used in the Scotland trial during the introduction phase to explain the study [4]. This message was reinforced by a postcard reminder about recyclable materials and direct community engagement with the target groups. In the Korean trial, budget constraints led to only introducing the trial via a presentation supported by the daycare association and limiting communication and engagement. The lack of communication material and engagement led to collection errors and confusion about diaper disposal sites and collection days.

The results of the two trials showed that establishing barriers and benefits to participation should precede and guide the introduction of a collection system. In the Korean trial, frequency of collections and acceptable odour management were important to participation. When assigning collection points, waste generation hotspots such as daycare centres increased quantities. Providing adequate and context-appropriate collection infrastructure raised participation. Appropriate communication to target groups foregrounding the environmental benefits of diaper collection schemes, such as in the Scotland trial, helped increase participation, social acceptance, and, with it, effective separation and collection.

As part of its global research activities, and in response to proposed government policy in South Africa, Kimberly–Clark Corporation, one of the major producers of AHP, contracted an independent research team to determine whether and how source-separated collections systems can work in low-income and resource constrained settlements.

In South Africa, used DDs are classified as general waste as long as they do not come from healthcare facilities. As such, they can be included with domestic waste in the municipality’s refuse collection services and disposed of at its landfill sites [21,25]. However, when municipal collections are limited or inadequate, used diapers may be discarded in public spaces, becoming a community health risk. This often happens in the many lower-income, high-density township housing areas where a number of tenants or ‘backyarders’ live in rooms or shacks on the property of the owner/ratepayer, who usually has only one municipal bin [18,26]. Bins are often stolen for other purposes, increasing the problem of informal waste dumping and its negative health effects.

## 3. Materials and Methods

### 3.1. Research Site

In 2022, the first and second authors conducted a pre-pilot research study to co-design a DD collection system for lower-income areas in townships and informal housing areas, using Community-Based Participatory Research [6]. This took place in collaboration with the community of Samora Machel township in Cape Town. Two important learnings came out of the process:

1. Mothers/parents preferred a cart service (pick-up) to collect used nappies from their houses for security and convenience reasons.
2. Similar to the Kim and Kim [4] study, Early Childhood Development (ECDs) centres were identified by the Samora Machel community as key partners as they are generators of diapers, provide access to parents with babies and could act as depot hubs for collections. Moreover, ECDs are seen as very important community structures in the townships, allowing mothers to go out to search for work or work and earn an income. ECDs in townships, according to Charman et al. [27], are regarded as micro-enterprises ranging from home-based childminders to informally established ECDs to fully fledged registered ECDs and supported by government and Non-Government Organisations (NGOs) or religious organisations. In the study on township economies in South Africa by Charman et al. [27], among the nine townships which formed part of their study, the ECD per township ranged between 6 and 42, with an average of 30 per township. Charman et al. [27] found that ECDs contribute to the micro-enterprise activities in the townships. On average, the owners were 53.7 years old, regarded as surrogate grand/mothers, and highly respected.

During June 2023, when the pilot collection programme was about to be implemented, a municipal refuse truck entering Samora Machel was held up by extortionist gang members demanding to be paid in exchange for protection to deliver refuse services [28]. Due to the increased security risk, the research team decided to exit Samora Machel township and find a safer site to implement the pilot project.

**Figure 1:**
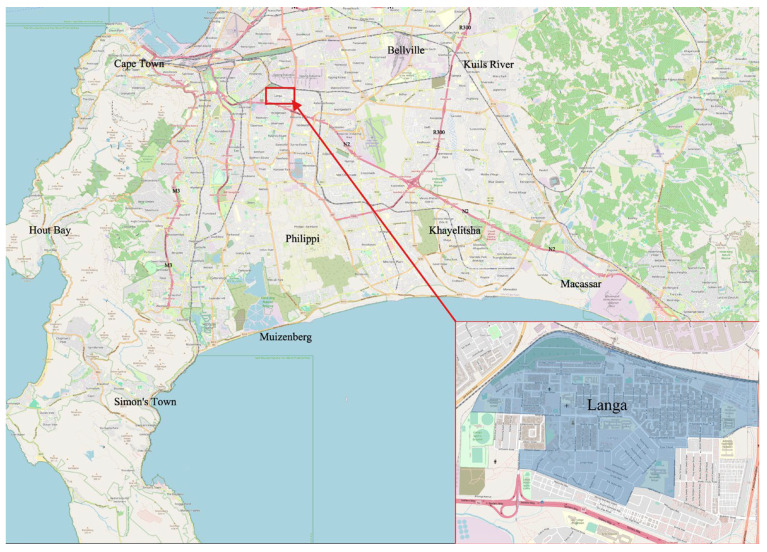
Location of Langa township, Cape Town.

Alternative sites were researched, and it was decided to move to Langa township (see Figure 1), where one of the project team members had helpful contacts. Langa is the oldest township in South Africa, and it is well-established with strong community organisations. It has a combination of formal and informal housing as well as ‘backyarder’ tenants. During the 2011 census, the population was reported to be 52,401. In 2019, it was reported as 62,000 [29] (At the time of the study, the 2022 census study was completed, but the data was not yet available).

**Figure 2:**
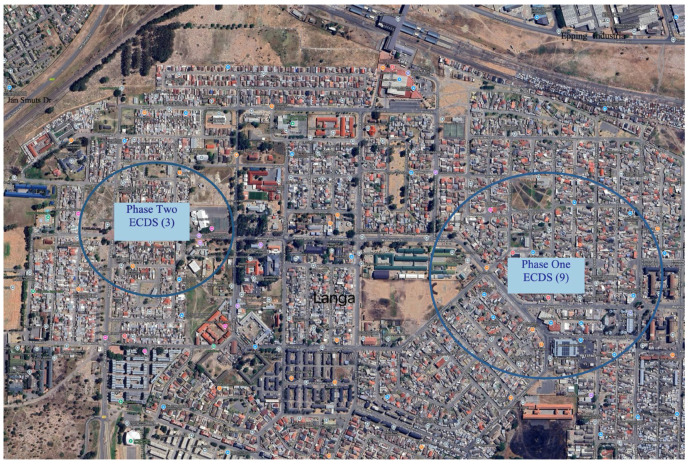
Areas in Langa where pilot studies were implemented.

The proposed collection programme was presented to the local councillor and the Langa ECD Forum, which comprised principals of the ECDs. They provided their input into the proposed collection systems and gave their approval for the pilot to go ahead. The pilot was implemented in two phases: Phase One with nine ECDs and Phase Two with three ECDs. In consultation with the ECD Forum, the following areas within the blue circles were identified as focus areas of the study (Figure 2).

The selection of participating ECDs was managed by the ECD Forum. Those keen to join and conveniently close to each other helped simplify the management of the project.

To implement and evaluate collection systems for DDs in Langa, a case study research methodology was used. The case study approach is particularly useful when there is a need to obtain an in-depth appreciation of an issue, event or phenomenon of interest in its natural, real-life context [30,31]. Stake [32] defines a case study as both the process of learning about the case and the product of the learning itself. Therefore, the case study approach lends itself well to capturing information on more explanatory ‘how’, ‘what’ and ‘why’ questions. In this study, we wanted to explore and describe ‘how’ the collection system was implemented and how it was received ‘on the ground’. Further, a case study offers insights into ‘what’ the learnings from the study are, what gaps exist, ‘why’ it existed in its delivery and ‘why’ one implementation strategy might be chosen over another [31]. 

Crowe [30] views Stake’s work as being particularly influential in defining the case study approach to scientific enquiry. Stake [32] defined case studies as intrinsic, instrumental and collective.

- An intrinsic case study is undertaken to learn about a unique phenomenon;
- The instrumental case study uses a particular case to gain a broader appreciation or understanding of an issue or phenomenon and
- The collective case study involves studying multiple cases simultaneously or sequentially in an attempt to generate a broader appreciation of a particular issue [33].

In this article, we describe a combination of an instrumental case study and a collective case study. Similar to Kim and Kim [4], two collection systems of disposable diapers were implemented sequentially (with some overlap) in separate areas to compare which would perform best in the Langa community.

Two collection methods were implemented during the study.

### 3.2. Collection System 1: Drop-Off Model (at ECD Centres)

In the drop-off method, a parent takes used nappies from home and drops them off at a central site for further responsible disposal. Similar to using ECDs in this study, daycares and schools have been investigated as institutional sites for source-separated collection of nappies by Kimberly-Clark in Australia, Brazil and the United States to understand how different types of collection systems fit within different local contexts [34,35,36].

Where municipal refuse collection services are irregular, such as in Langa, the free twice-weekly used diaper collection service offered by the project was welcomed by the ECDs. In consultation with the ECD Forum, nine centres came forward and were signed up for the programme. From these nine ECDs, 126 parents with babies using disposable diapers signed registration and consent forms. The following information was provided to the parents prior to signing up:

- ECD principals first informed parents verbally of the project and encouraged them to register and join in the collections.
- Researchers produced posters for the ECDs, which explained the reasons for the project and how to participate.

A starter pack was provided for parents. This included a small 12-L (370 mm high) pedal bin with bin liners as well as a sling bag (400 × 400 mm) to take nappies and drop them off on Tuesdays and Fridays at the ECD when they left their infants before going to work.

**Figure 3:**
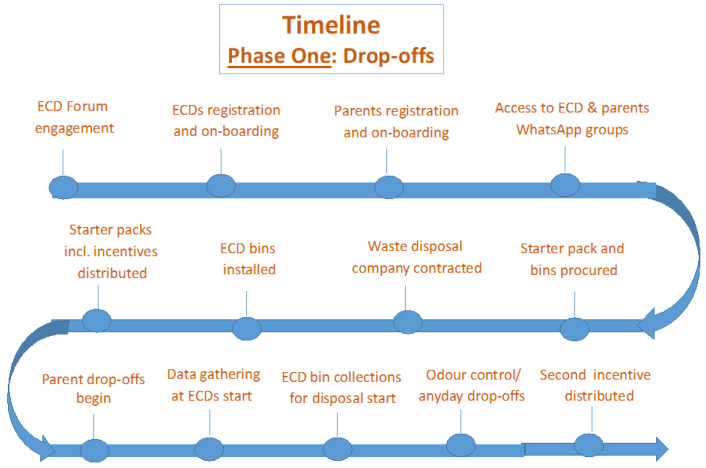
Timeline for phase one: Drop-off model.

For the ECDs, a large wheeled bin (240-L) was provided and placed away from the children’s area. A private company accredited by the municipality to collect and transport medical waste was contracted to take the diapers from ECDs to the municipal landfill for safe disposal—the only safe option currently available in the Cape Town area. The timeline for Phase One is illustrated in Figure 3.

After three months, the first phase of the project was operational, and the second collection model could be launched.

### 3.3. Collection System 2: Pick-Up Model (Collection from Homes)

The ‘pick-up’ method was an outcome of the co-designed pre-pilot research, which showed parents’ preference for door-to-door collections due mainly to township safety and security concerns [6]. The pick-up method was also the preferred method in the Scotland study [4].

**Figure 4:**
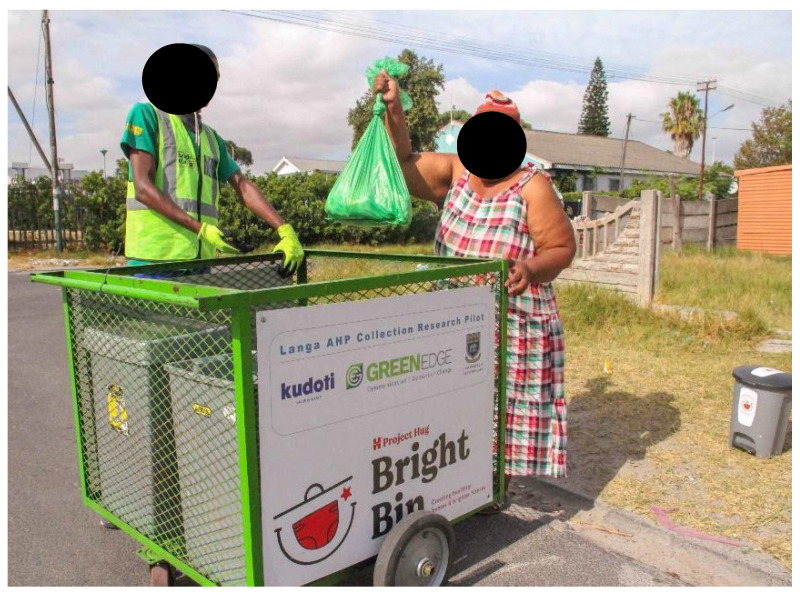
The cart collector is picking up nappies from a parent.

The pick-up model employed collection teams with push carts to pick up used diapers from parents at their homes in surrounding streets and take them to the ECDs. Figure 4 shows how the carts and collectors were branded for identification as legitimate. Three ECDs and 40 parents with babies were registered. Parents were also provided with starter packs, which contained the same items as those for Phase One. Those who wanted to use the sling bags to take used nappies from home to the ECD could do so.

The Langa Safety Patrol (LSP), a community-based organisation, agreed to recruit and oversee six young adults to manage cart collections as part of their rehabilitation programme. This avoided perceptions of favouritism by the researchers and showed evidence of community upliftment by the project. The collectors were given overalls and gloves, trained to handle push carts and paid a daily stipend to pick up used DDs from homes. Large bins were provided to participating ECDs for collected waste, and a waste company was contracted to dispose of used nappies at the municipal landfill.

Street addresses of the registered parents were provided to collectors. However, coordinating with households for someone to be on hand for pick-ups during the day was problematic. This was partly because many registered parents would have been at work.

Yet, with prominent branding and signage, the collection carts soon gained the interest of parents in the area who were not registered with an ECD but also wanted their used nappies to be picked up. The researchers acceded to this, but the result was that it became impracticable to track how many non-registered parents were participating on any collection day. This differed from Phase One, where registered parents only were being recorded. It also meant that the collection of nappies per parent/child between Phase One and Two could not be directly compared.

**Figure 5:**
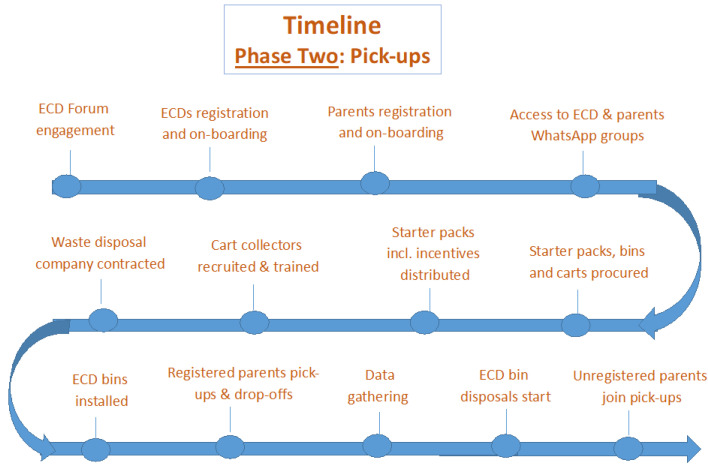
Timeline Phase two: Pick-up model.

The timeline for Phase 2 is illustrated in Figure 5.

### 3.4. Communication and Engagement

The brand name “Bright Bin” and logo were developed (See Figure 4) to provide credibility and trust and encourage greater participation by participants in collections. The Bright Bin project was indicated as being sponsored by Huggies. Overall, there was positive recognition of the branding and its support for the project because it addressed ECDs and parents’ concerns about public health risks. This was emphasised in the communications message framing: “Join a research project to find better ways to manage nappy waste and reduce sickness”.

Information leaflets were included in the starter packs, and posters were distributed for installation at the ECDs as regular reminders. “Nappies only” decals on household nappy bins discouraged other waste from being mixed up in bins. All material was printed in English and isiXhosa, the home language of most residents in the township [29].

A shopping voucher was included in parents’ starter packs as a contribution for their time and effort and an incentive to reciprocate by participating. The incentive was not communicated before parents signed up.

With permission from the parents, the ECD principals opened access to parents’ WhatsApp groups, and voice notes became the main channel for information sharing. The overall encouragement and support by ECD principals and staff for parents to join in and participate was a major factor in the pilot meeting its objectives.

### 3.5. Data Collection Methods

When applying a case study method, it is important to use multiple data collection methods to enhance the validity of the results [30,31,32,37]. The following data collection methods were used:

#### 3.5.1. Measuring the Mass of Material Collected

**Figure 6:**
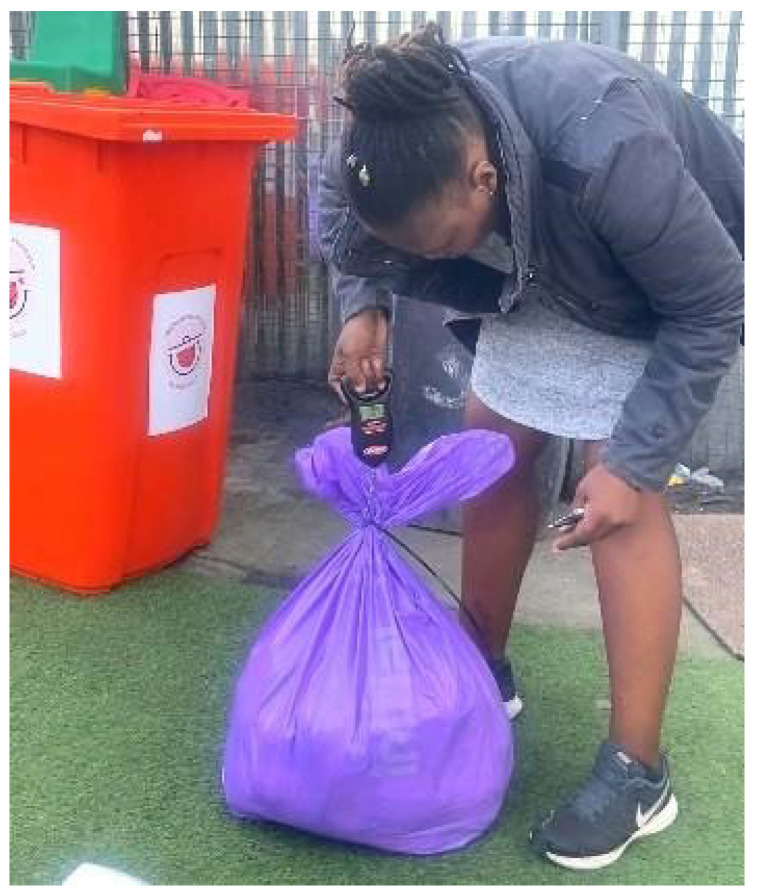
Measuring mass data with a digital weighing scale.

The mass of diapers generated at the ECDs and brought in by parents and cart collectors was weighed on collection day, twice weekly, by the trained community research team (see Figure 6). This data was uploaded to a web-based platform where it could be independently verified. The data could then be tracked and analysed over the duration of the project to determine the performance of the two models.

#### 3.5.2. Progress Observation and Reporting

Research team members were in close contact with parents and ECD principals via a WhatsApp group. They reported weekly on the progress of the drop-off and pick-up collection models. Weekly reports were provided to project leaders.

#### 3.5.3. Fieldwork Survey

An independent research company was contracted in November 2023 to conduct a survey and focus groups with parents and principals to document their experiences and feedback on the project. The research company also followed up with parents who dropped out of the pilot projects. In total, the research consultants interviewed 28 parents, three principals and three collectors.

#### 3.5.4. Focus Group Discussion

Two focus group discussions were facilitated to ascertain key enablers and barriers in the pilot studies. The first focus group was held with the ECD principals, and the second with the pilot research team.

#### 3.5.5. Illegal Dumping Mapping

During July and August 2023, illegal dumpsites in the Langa area were identified and mapped by capturing their GPS coordinates, with the types of waste identified. The prevalence of DDs in the dumpsites was a particular focus. The data was then used to generate a map of all dumpsites in the area in relation to the ECD centres. While mapping, the fieldworkers (authors 4 and 5) made observations and had informal discussions with community members to understand dumping practices.

The pilot project concluded at the end of November 2023, and a report-back session was held on 8 December 2023, with stakeholders from the community, municipality, and funders. The results and lessons learned from the pilot study are as follows.

Note: There were two research teams in this study, which will be referred to as the Langa research team and the external research team. The Langa research team consisted of three researchers from LEAPs, a local community-based non-profit organisation that assisted with the day-to-day operations of the pilot project. Of the external research team, author 1 of the article was the lead researcher, while author 2 initiated and directed the overall project.

## 4. Results and Discussion with Lessons Learned

### 4.1. Both Collection Models Fulfil a Need

**Figure 7:**
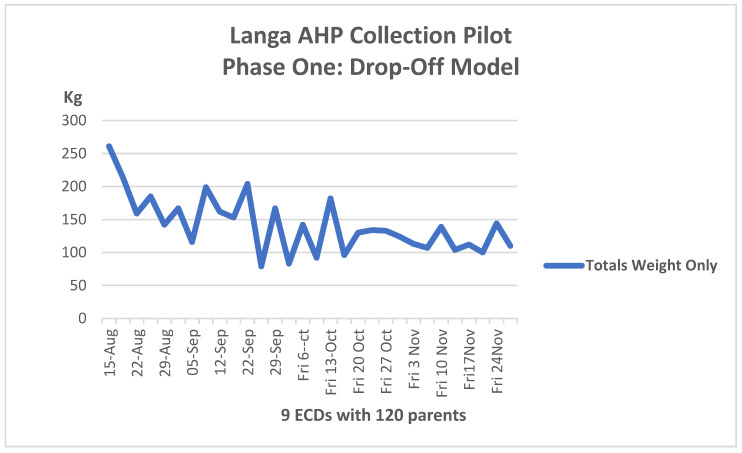
Total weights collected weekly during Phase 1-drop-off model 15 August–28 November 2023.

**Figure 8:**
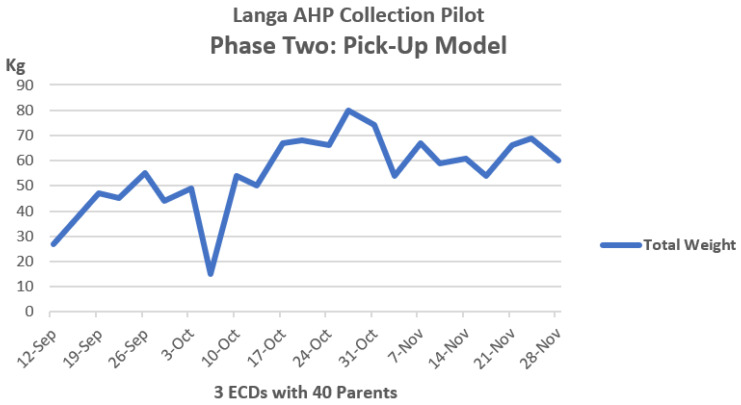
Total weights collected weekly. Phase 2- pick-up model 12 September–28 November 2023.

In both the drop-off and pick-up pilot projects, households diligently separated DDs from the rest of the household waste. By the end of the project, nearly two tons of nappies were being consistently collected monthly from 12 ECDs. Figure 7 and Figure 8 provide an overview of the weight of DDS collected.

Interestingly, the drop-off model tapered down until it reached a plateau, while the pick-up model showed an upward trend.

It became clear that a mixed model of collections would perform best in low-income communities like Langa. Working parents who drop children off at the ECD found it convenient to drop off their DDs. Parents who are unemployed or stay at home and whose children do not attend ECDs benefit from the pick-up collections.

While twice-weekly collections were made available, due to DD odour problems, parents asked if they could drop off bags on any weekday. This was acceded to by the ECDs, which helped increase volumes and reduce dumping. Adaptability to the local context, as well as accessibility and convenience, became key factors in maximising collection volumes.

### 4.2. Key Role of ECDs

As was suggested during the co-design phase in Samora Machel [6], in Langa, and in Kim and Kim [4], ECDs were identified as key role players and partners in collection projects, as significant generators of nappies themselves and as key respected support structures in townships [27].

The initial presentation to and subsequent collaboration with the Langa ECD Forum paved the way for its member ECDs to support the pilot. ECD principals and staff then assisted the research team in encouraging parents to sign up and generally champion the project with parents, colleagues and the community.

The Langa research team noted how impressed they were by the commitment from some of the principals as they realised what benefits the project could have for the Langa community. At the beginning of the project, some of them went out of their way to assist parents in participating. The following was shared by a principal:

“I went to each of my parent’s households to see why they weren’t bringing nappies…. I stand by the gate on Tuesday mornings to check if parents are bringing and ask them to go back and fetch their nappy bags.”

In this project, they “bought” into the project with enthusiasm, partially because they co-designed its implementation. In future projects, ECDs should be part of the co-design of the planning of the project as well.

Important to note that in the Korean study [4], the daycare staff were burdened with nappy separation. This pilot project avoided extra labour for the ECD staff, except for the principals’ initiatives to motivate parents.

### 4.3. Importance of Starter Packs

**Figure 9:**
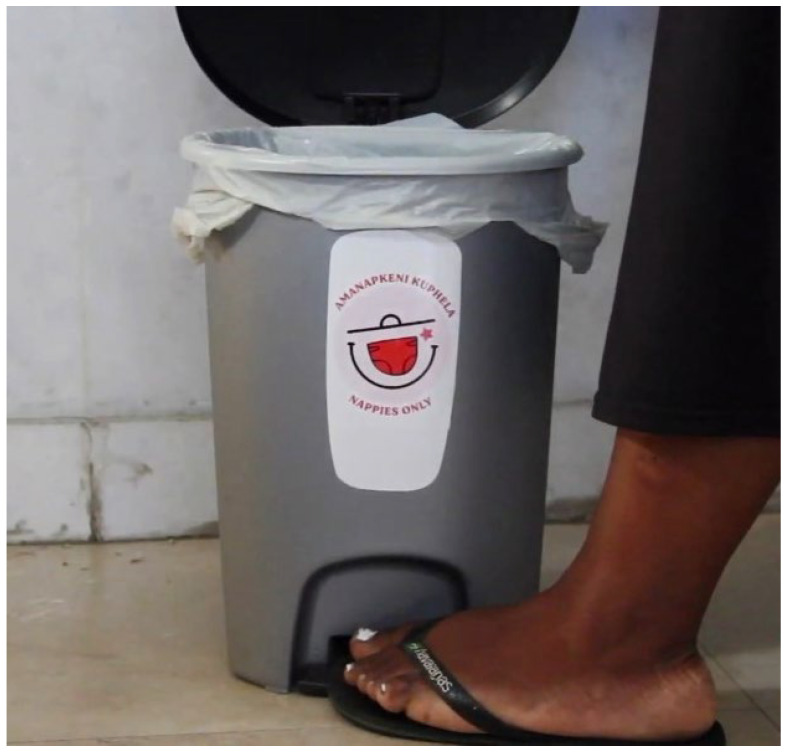
Pedal bin for holding used nappies at home.

Convenience is essential to encourage a behavioural change to a new household practice [37]. Therefore, a ‘starter pack’ for parents was assembled to make it easier for them to participate in collections. Each parent was given a small pedal bin (Figure 9) with bin liners to gather used nappies at home. Included was a large sling bag for those taking nappies and dropping them safely off at their ECD.

An information leaflet explained why and how to participate. A sanitiser, some new diapers and a shopping gift voucher were also included. These starter pack items conveyed ‘customer care’, respect and dignity and increased the probability of reciprocity.

To help ensure that other kinds of household waste did not go into the nappy bin and distort weight data, ‘Nappies Only’ decals were applied to the bin top and side as previously described in English and IsiXhosa.

The care and attention to detail of the starter packs also helped convince sceptics to take the project seriously. The following was noted by a research team member:

“In one ECD, the Principal wouldn’t let us have access to parents at all … it took us a while to build trust, and when we delivered her parent starter packs, only then she actually gave us access to the parent body. The starter packs made clear the reality of the project and so helped gain her trust.”

Not long after the start of collections, we received information that some parents were complaining about odours coming from the nappy bins. Small mesh bags containing pellets of activated carbon to keep inside bins were distributed to the parents. This seemed to address the nappy odour problem as complaints receded. Activated carbon pellets can be included in future starter packs.

### 4.4. Reflections on Getting Started

Kim and Kim [4] mentioned in their discussion of the Korean diaper collection projects that the study was too short for the participants to adapt to the routine of the collection system.

In the drop-off model of the Langa study, it took some time for the parents, despite being motivated, to get used to the new system of bringing their DDs to the ECD. Parents apologised, stating that it is a “new thing to do”, and they are simply forgetting to take diapers with them.

“I had already packed the bag, and in the morning rush I simply forgot it [the red bag] by the door when leaving”.

“We will get used to it, and then it will become normal”.

WhatsApp groups were formed with the consent of the parents, principals and community researchers to send reminders via WhatsApp voice notes. Visits from principals also assisted with the start of the project until it became part of the household routine.

In the pick-up model, there was an early high level of participation, which grew as the visibility and convenience of the carts gained increasing attention.

### 4.5. Frequency of Collections

As mentioned above, early on in Phase One of the pilot (drop-off model), parents requested that they drop off DDs on any weekday instead of Tuesdays and Fridays only, which the ECDs readily agreed to. Due to odour issues, this particularly affected families who were living in small dwellings or apartments, as this quote from a parent attests:

“The smell gets really bad on the weekend in the house, we live in the flats.”

The drop-off facility’s importance was confirmed by parents who indicated that they either do not have bins to store their nappies outside the house, do not have space for bins, or crime made it impossible: “I can’t put the bin outside, it will be stolen”.

### 4.6. Benefits of Collections by Mobile Street Carts

Township life can be dangerous, particularly for women. In the pre-pilot research, interviewees said they would prefer diaper collection by carts from their homes rather than taking them to the ECD and dropping them off there [6].

However, parents registered at the ECD worked during the day, so they were not at home when the carts were on their Tuesday and Friday morning collection rounds. Ways were tried to streamline pick-ups from houses of ECD parents absent at work when the pick-up cart came by, such as giving them hooks to put on their front gates on which to leave bags for collection, but this was not taken up. Most homes are intergenerational; however, so often, a grandmother, aunt, or uncle would be at home to hand over their diaper bag to collectors.

The high visibility of the carts roving through the streets with Bright Bin signage attracted attention from residents. More and more parents began to ask to join in, which they readily agreed to.

The carts also communicated the project’s socio-economic value to the community, as it was seen to be creating much-needed income for young people and helping in their rehabilitation.

The carts attracted the attention of a local TV journalist who produced a short video of the nappy project, which was broadcast on national television. This was well received by the community as it portrayed Langa in a positive developmental light. It also contributed to the status of the project amongst parents and helped maintain their commitment to it.

### 4.7. Motivation for ECDs, Parents and Collectors to Participate in the Project

During the focus group discussions, various motivations were expressed for stakeholders’ participation in the project.

#### 4.7.1. ECD Principals

Some of the factors for participating in the project have already been shared. ECD principals further felt that Langa is very dirty (as can be confirmed by the images) and that nappies are dumped. They believe the project can contribute to a cleaner environment, particularly to prevent blocked toilets and drains caused by dumped nappies.

**Figure 10:**
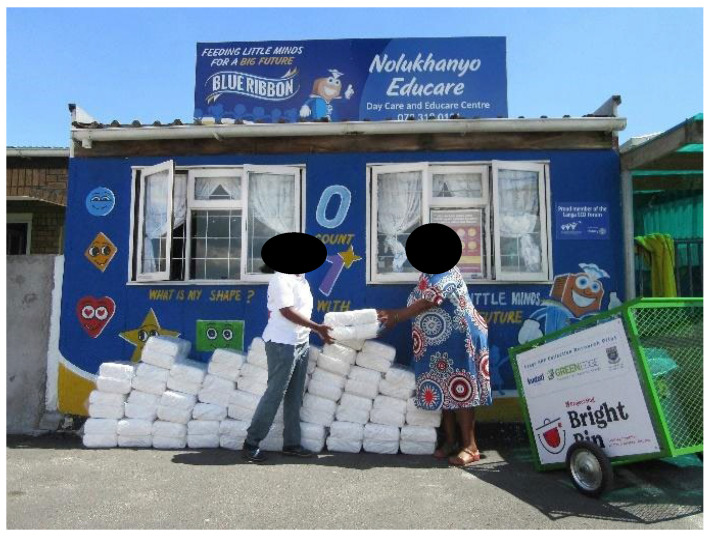
Packs of DD are being donated for distribution to ECDs.

The ECDs were given a bulk donation of new DDs (Figure 10) to assist with the caring of the babies for the ECDs in each area, as well as a R200 shopping voucher. The donations and vouchers acted as an incentive to support the project. ECD principals mentioned that the donations were valued as they could be used for infants whose parents struggled to afford disposable nappies. During the pilot study, ECDs had fewer babies with rashes, as there were sufficient nappies for regular changes during the day.

The high level of motivation of the ECD principals in promoting the project was described by a parent: “Yes, she (ECD principal) reminds us of the nappy collections on Tuesdays and Fridays and at the beginning she would come to our place to make sure we are doing it right”.

#### 4.7.2. Parents

Of the 28 parents interviewed, 83% said their motivation for participation was that they want a cleaner and healthier environment for their children. They provided examples of undesirable disposal consequences: DDs in Langa are “being dumped and then eaten and dragged by dogs and rats”. Dumps are breeding grounds for rats and maggots. Parents raised health concerns when their children played on the illegal dumpsites containing used DDs.

Each registered parent was given a R100 (5.00 USD) shopping voucher as a ‘forward incentive’ and appreciation for their participation in collections. This might have been an initial motivation to participate. However, their widespread appreciation of the positive impacts of the project being a cleaner, healthier environment seems to be a stronger incentive to continue participating. This is also evident in that some parents indicated that they had encouraged other parents in their ECD to join in collections. Parents not registered with an ECD who did not receive incentives also joined the project, confirming perceptions of its value to general community health.

#### 4.7.3. Collectors

The main motivation for the cart collectors to participate was the opportunity to earn an income. During the interviews, the collectors described their work as enjoyable; it gave them a sense of purpose, they gained credibility amongst the community by providing a public service, and they had an opportunity to educate the community about responsible waste management. Working under the supervision of the LSP enhanced their standing and helped ensure the safety of the community.

### 4.8. The Role of Branding in Encouraging Collection

While it can have good intentions, a private sector-funded research intervention into a low-income, high-density township may be viewed initially with caution by the community. There are also cultural issues in African communities when dealing with the disposal of bodily fluids.

A range of ways were employed to address this when engaging with the Langa community to help achieve the pilot’s aims of optimising collection quantities. One of these was to create general awareness that it was supported by a well-known and trusted brand.

In interactions and in the surveys with the participants, there was a high level of positive awareness of the Huggies brand. In studies by Schenck et al. [6] and Acker-Cooper et al. [7], Huggies was identified by the communities involved as a respected brand, although the colloquial and generic reference to DDs is “Pampers”.

The widespread acceptance of the pilot and participation in collection activity suggests a correlation between brand trust and exposure, as well as community engagement in collections. It also points to the useful role of brands and branding in encouraging collection behaviour change as a topic for further research.

### 4.9. Synchronising Collection Logistics

AHP waste, unlike other waste materials, cannot be left in one place for too long. A critical component of the project, therefore, was to synchronise collection logistics from households (by drop-offs or pick-ups) to ECDs and from there (by the waste contractor) to landfill regularly and effectively. Regular collection was critical for the community to trust the project.

If the contractor does not collect the nappies at the expected time, children and ECD staff are exposed to excessive bin odours—especially on hot days. Also, on collection days, weighing of accumulated material at the ECDs must take place soon after the collection carts have deposited their loads at the ECDs and before the waste contractor comes to collect for transfer to the landfill. So careful synchronising of timing is required.

### 4.10. Disposal of AHP Diaper Waste to Landfill

In South Africa, diaper waste that does not come out of healthcare facilities is classified as general waste and so can be accepted at municipal landfills, provided there is a negotiation between the municipality and waste generator [25]. Municipalities are, however, generally cautious about accepting accumulated volumes of AHP diaper waste at landfills, and as yet, there are no norms and standards to guide them.

At the outset of the project, the research team engaged with the City of Cape Town to collaborate and assist with disposal at municipal landfills. However, this was declined. Private waste companies were then contracted to transport the collected nappies from the ECDs to a landfill co-owned by the municipality and the contractor.

### 4.11. Mapping Informal Dump Sites

**Figure 11:**
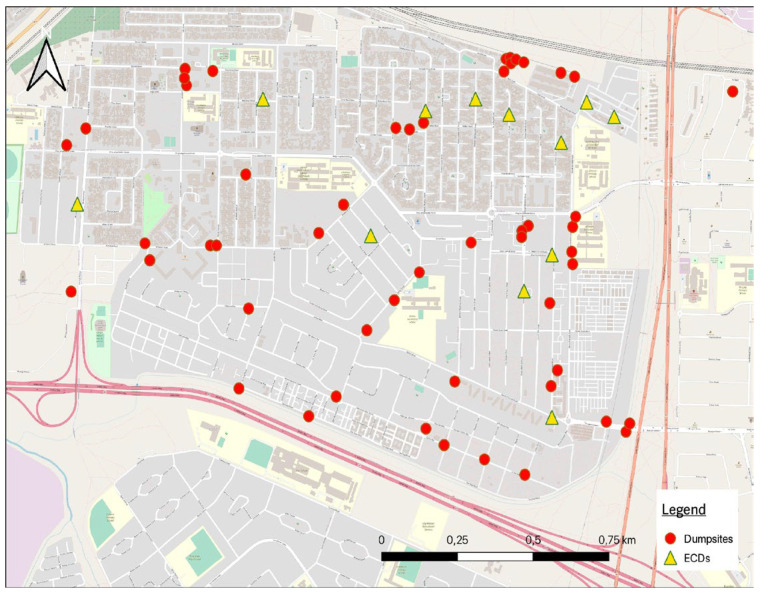
The Langa map indicates the illegal dumping in relation to participating ECD centres.

**Figure 12:**
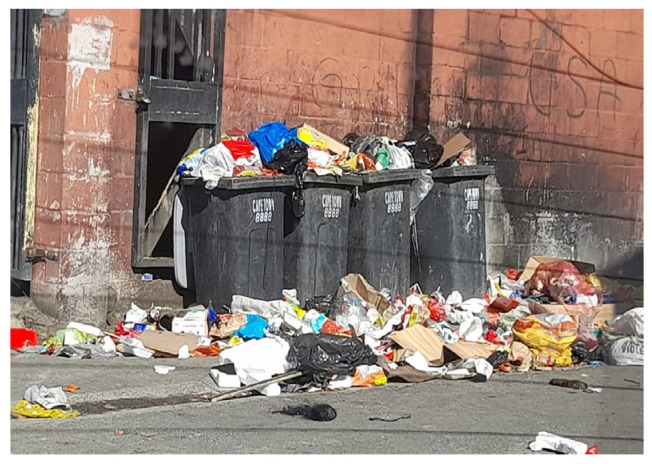
Overflowing waste bins in Langa.

Significant dumping in Langa, including nappies, amongst other waste types, can be seen in Figure 11 and Figure 12. Over 50 illegal dumpsites were found in Langa. The dumpsites were visible between the houses and in open spaces, creating health risks for residents, children and the environment.

In addition to dumpsites, the fieldworkers also provided photos (see Figure 12) showing overflowing bins. These show that the provision of waste infrastructure and the collection frequency are insufficient—which results in dumping. This often happens in the many ‘backyarder’ and informal housing areas in South Africa, such as the Langa pilot sites. In these areas, a number of tenants live in rooms or shacks on the property of the owner/ratepayer, who is allocated only one municipal bin [26]. This bin soon overflows, so tenants have little option other than to discard their household waste, including DDs, in open spaces, which become dumpsites.

In an area referred to as the ‘Durbanville (Durbanville is a high-income area in Cape Town) of Langa’, the fieldworkers were “struck by the absence of illegal dumpsites”. One of the research team members explained that this area has bigger houses and plots and receives better waste management services as the residents are paying rates. They receive “wheelie bins (Wheelie bins are garbage bins with wheels and handles. Available in 130l or 240l see Figure 12)” and have a yard in which the bin can be safely secured.

During the mapping process, disposable nappies were observed as a waste fraction present in all the dumpsites. Also, according to the fieldworkers, the main reason for blocked drains is dumped DDs, which is similar to what was found in Samora Machel [6].

### 4.12. Criminal Activities

In addition to insufficient waste services and infrastructure, criminal activities also account for dumping. While busy with mapping, fieldworkers observed a spaza shop (A Spaza shop is an informal convenience shop in South Africa’s townships) owner dumping cardboard boxes on the roadside. When questioned, he reported that he pays ‘protection’ fees to a gang, and failure to allow him to dump could result in retaliation from the gang.

It was shared that petty criminals steal municipal refuse wheelie bins from households and businesses and then resell them at a nominal fee. Residents say they prefer not to put their bins out on municipal collection days for fear of bins being stolen while they are at work. Those who have had bins stolen have few options but to dispose of their waste by dumping it. Re-applying for a municipal wheelie bin can be a lengthy process, so residents resort to buying stolen bins, fuelling a cycle of petty crime. Bins of those who have them overflow through not being collected, and they pay the same petty criminals to go and dump illegally. This practice was also confirmed by Schenck et al. [6] in Samora Machel township and in studies of the development of an AHP collection system in Durban, South Africa, where precautions had to be taken to prevent criminal activities [38].

Bright, colourful bins were issued to ECDs during the research pilot and tied to poles and gates outside some ECDs to prevent them from being stolen. Other ECDs put their bins outside in the morning for easy access and took them back inside at lunchtime. Coloured yellow or orange to differentiate them from municipal black bins, the community recognised ECD bins as those from the collection project, and none were stolen.

### 4.13. Cultural Beliefs

As was found in the Samora Machel and other studies, Refs. [6,7,9,19,39] cultural beliefs around body fluids/faecal matter were a sensitive subject. According to the Langa research team, “parents keep asking what we are going to do with their babies’ faecal matter”. They believe that it might be used for practices that may harm their child. Cultural beliefs are therefore important to explore and address. The Langa research team dealt with the issue by being honest and open about the project and its aims.

### 4.14. The Importance of Social Media and Communication

An aspect raised by all participants was the importance of regular communication between all the role players in the pilot project. WhatsApp voice notes were the main social media channel used by the team to share information with parents. Parents emphasised the role ECD principals played in encouraging and reminding them via WhatsApp messages. Parents also expressed appreciation for principals who visited those who did not have smartphones and needed to be kept informed.

### 4.15. The Value of Community-Based Organisations (CBOs)

Three trusted Community-Based Organisations in the Langa community were key role players in the implementation of the pilot project:

- LEAPs, an education and social development non-governmental organisation (NGO), was sub-contracted as a pilot project partner and operations manager.
- Langa ECD Forum, a well-established and competent organisation, played a key role in accessing ECDs and fostering participation by parents;
- Langa Safety Patrol (LSP) grew out of the local community policing forum. It gave the project greater standing and protection in the community, so reducing security risks. LSP recruited and managed the cart collectors, which lightened the administrative burden of the implementation team.

### 4.16. Unintended Positives of the Project

During the focus groups and interviews, parents emphasised the benefits the project was making to the environment, remarking that less dumping of nappies was visible, as well as fewer blockages of sewerage systems.

There was an increased sense of sharing and working together for a cleaner environment, which helped with community cohesion. Parents also shared an understanding of the role that communities can play and the responsibility they can take for a cleaner and healthier environment. Parents also said that if the community started to take care of their environment through such projects, this would teach children to take care of their waste and the environment as well.

### 4.17. Exploring the Longer-Term Sustainability of the Project

After the research pilot was completed, a meeting was held to report back to community and municipality stakeholders on the project outcomes and ways forward. This included showing a video of the project (6 min), accessible at: https://www.youtube.com/watch?v=XURskN_Enp0 (accessed 7 May 2024).

A general call came from the community for the nappy collection system to continue. With bins and carts infrastructure still in place, this is happening at a low-cost level. Opportunities for its continuation are being sought by role players guided by questions regarding longer-term sustainability:

- How can financial sustainability be ensured with or without support from the municipality, or how can low-income communities be serviced appropriately? [38,39,40]
- How can environmental sustainability be ensured if all other household waste is not removed regularly but is still being dumped?
- How can social sustainability be ensured? Can the community take responsibility for ensuring a cleaner environment and work hand in hand with the NGOs to develop waste removal systems relevant to their context?
- What alternative viable waste management systems can be developed for informal housing areas [41,42]?

## 5. Limitations of the Study

In terms of the implementation of the novel disposal practices where the drop-off model was concerned, participation did not commence immediately. Future studies should take into account the preparation time necessary to implement new routines.

In terms of the pick-up model, coordination with households to arrange pick-ups presented a challenge, and participants did not accept a low-cost, practical solution. The hooks that were provided were not utilised on the gates of participating households. It is not clear whether the refusal of households to make use of this alternative was due to cultural taboos, repurposing of provided hooks for other purposes or other unidentified reasons. Solutions need to be co-designed at all times. 

Not all participants had access to smartphones, and therefore, more than one mode of communication was needed, and principals compensated for this by visiting the parents.

A comparison of phases one and two of the study in terms of collection was affected by the participation of unregistered parents on collection days. These parents’ participation could also not be tracked on collection days.

## 6. Conclusions

The article describes a case study of two DD collection models that were tested in low-income subsections of the Langa township in Cape Town. The ‘drop-off’ model, where parents took DDs from home and deposited them at ECDs, performed well at the start, then tapered into a plateau. The ‘pick-up’ model using mobile carts steadily increased collections partly due to additional parents joining in who were not registered with an ECD and took advantage of its door-to-door convenience. A combination or ‘hybrid’ of the two models has the potential to engage participants with differing economic resources since it provides safer diaper disposal opportunities for both working and unemployed parents. A hybrid model would also increase the visibility of the project to other community members outside the sphere of the ECDs. ECDs were shown to work well as communal collection hubs and were key partners in motivating parents to participate. Safety risks were reduced by bringing in the local community policing forum to provide some security.

Overall, the study confirmed that families in LMIC would separate DDs from other household waste and make them available for collection, particularly if it was made easy for them to do so. Measures that were conducive to this end included sustained motivation and reminders to participate, clear communication of the benefits and aims of the disposal scheme, leveraging trusted branding and the provision of accessible instructions and disposal resources.

The study confirmed that DDs are difficult to manage for households living in low-income, high-density areas, and many diapers are dumped due to the unavailability of efficient waste management services [6,38,39]. Safety and hygiene concerns, storage challenges, crime and cultural taboos contribute to undesirable disposal practices.

The pilot study provides initial evidence that a convenience-based, community-based, and private sector-led collection system can support households (with various financial constraints) in disposing of DD waste responsibly instead of dumping it in open spaces. It indicates how support for diaper disposal systems can be enhanced by involving community institutions, providing viable short-term storage options, harnessing sustainable communication channels and leveraging trusted branding. It is recommended that this pilot study should be followed by a main study extending the geographical scope and time of the interventions.

Policy options such as Extended Producer Responsibility [43] can support municipalities and communities to co-design tailormade options for a cleaner and healthier environment.

## Data Availability

Data is available from the authors.
